# Ethanolic Extract of the Fungus *Trichoderma asperelloides* Induces Ultrastructural Effects and Death on *Leishmania amazonensis*

**DOI:** 10.3389/fcimb.2020.00306

**Published:** 2020-07-15

**Authors:** Danielle de Sousa Lopes, Uener Ribeiro dos Santos, Danielle Oliveira Dos Anjos, Lauro José Caires da Silva Júnior, Vanderlúcia Fonseca de Paula, Marcos André Vannier-Santos, Izaltina Silva-Jardim, Thiago Castro-Gomes, Carlos Priminho Pirovani, Jane Lima-Santos

**Affiliations:** ^1^Departamento de Ciências Biológicas, Universidade Estadual de Santa Cruz—UESC, Ilhéus, Brazil; ^2^Laboratório de Produtos Naturais, Universidade Estadual Do Sudoeste da Bahia—UESB, Jequié, Brazil; ^3^Laboratório de Inovações em Terapias, Ensino e Bioprodutos, Instituto Oswaldo Cruz, Fundação Oswaldo Cruz-FIOCRUZ, Rio de Janeiro, Brazil; ^4^Laboratório de Biologia Celular e Parasitos Intracelulares, Universidade Federal de Minas Gerais—UFMG, Belo Horizonte, Brazil

**Keywords:** *Trichoderma*, chemotherapy, leishmanicidal, leishmaniasis, *Leishmania amazonensis*

## Abstract

The *Trichoderma* genus comprises several species of fungi whose diversity of secondary metabolites represents a source of potential molecules with medical application. Because of increased pathogen resistance and demand for lower production costs, the search for new pharmacologically active molecules effective against pathogens has become more intense. This is particularly evident in the case of American cutaneous leishmaniasis due to the high toxicity of current treatments, parenteral administration, and increasing rate of refractory cases. We have previously shown that a fungus from genus *Trichoderma* can be used for treating cerebral malaria in mouse models and inhibit biofilm formation. Here, we evaluated the effect of the ethanolic extract of *Trichoderma asperelloides* (Ext-Ta) and its fractions on promastigotes and amastigotes of *Leishmania amazonensis*, a major causative agent of cutaneous leishmaniasis in the New World. Ext-Ta displayed leishmanicidal action on *L. amazonensis* parasites, and its pharmacological activity was associated with the low-molecular-weight fraction (LMWF) of Ext-Ta. Ultrastructural analysis demonstrated morphological alterations in the mitochondria and the flagellar pocket of promastigotes, with increased lipid body and acidocalcisome formation, microtubule disorganization of the cytoplasm, and intense vacuolization of the cytoplasm when amastigotes were present. We suggest the antiparasitic activity of *Trichoderma* fungi as a promising tool for developing chemotherapeutic leishmanicidal agents.

## Introduction

Fungi of the genus *Trichoderma* are endophytes, well-known as pathogens for other fungi (Atanasova et al., 2013). Because of their high reproductive rate, their ability to secrete antibiotics and biomass-degrading enzymes as well as to colonize crop species, they play an important role in agriculture and biotechnology (Schuster and Schmoll, 2010).

The *Trichoderma* metabolites with antibiotic activity are distinguished by molecular weight and polarity: (a) high molecular weight and polar molecules, such as peptaibols, have direct activity on their antagonist membrane; (b) low-molecular-weight molecules and volatile compounds, including polyketides, such as butenolides and pyrones, have important pharmacological activity (Cardoza et al., 2005).

Reports on the antiprotozoal activity of *Trichoderma* fungi are scarce. It was demonstrated that hirsutellone, an alkaloid dimer obtained from *Trichoderma* sp. and trichothecenes from soil-derived *Trichoderma brevicompactum* exhibited interesting antimalarial activity upon *Plasmodium falciparum* (Isaka et al., 2006; Klaiklay et al., 2019). Interestingly, (Podder and Ghosh, 2019) showed a possible indirect control of malaria when observing the efficacy of the fungus *Trichoderma asperellum* against *Anopheles* spp. larvae, demonstrating a novel application of fungal constituents as anopheline malaria vector control agents. Iwatsuki et al. (2010) reported that trichosporin B-VIIa and trichosporin B-VIIb peptaibiotics, produced by *Trichoderma polysporum*, exerted antitrypanosomal activity against *Trypanosoma brucei*, suggesting that this compound interacts in the protozoan membrane. However, there are no reports of the activity of *Trichoderma* metabolites against other neglected diseases such as leishmaniasis, which comprises a serious public health problem.

There are few therapeutic alternatives for neglected tropical diseases largely due to the pharmaceutical industry's lack of interest in developing new drugs. Thus, new chemotherapeutic agents are urgently required (Rajasekaran and Chen, 2015). Natural products have been an important focus in research for new drugs, mainly aiming for more efficient treatments for infectious diseases caused by parasitic protozoa such as leishmaniasis (Tenguria et al., 2011).

Around 700,000 to 1 million new leishmaniasis cases occur annually in 98 countries, and 350 million estimated people are under infection risk in endemic areas (World Health Organization, 2020). Infection begins with the blood meal of *Phlebotomus* (Old World) and *Lutzomyia* (New World) sand flies. The parasites have a digenetic life cycle, requiring the phlebotomine vectors that harbor the promastigote, flagellar form in its gut and the vertebrate mammalian host in which the non-motile amastigote form (Vannier-Santos et al., 2002). The promastigote forms infect macrophages, and monocytes differentiate into amastigotes that proliferate intracellularly and endure the host immune response causing the disease. These disease clinical manifestations range from asymptomatic lesions to death, depending on the species and the host's immunity (Scorza et al., 2017).

Currently, pentavalent antimonials, paromomycin, pentamidine, amphotericin B, and miltefosine are some of the medications used in the treatment of leishmaniasis (Akbari et al., 2017; Burza et al., 2018). However, these drugs are administered for a prolonged period and have high toxicity, which normally lead to interruptions in the treatment, thus contributing to parasite resistance and the parasite favoring pathogenicity (Maltezou, 2010; Olliaro, 2010; Matrangolo et al., 2013). These drugs may inhibit both glycolysis and β-oxidation of fatty acids, alter membrane permeability, and inhibit protein synthesis (Rajasekaran and Chen, 2015). In the present work, we have shown the effects of the treatment with the ethanolic extract of *Trichoderma asperelloides* on *Leishmania amazonensis* parasites, which makes this fungus an innovative alternative to the development of new leishmanicidal drugs.

## Materials and Methods

### *Trichoderma asperelloides* Cultures and Preparation of the Ethanolic Extract

The *T. asperelloides* strain LIBASP02 was cultivated in potato dextrose agar (PDA) in Petri plates at room temperature for 10 days. The crude ethanolic extract from mycelium and spores was obtained from washing cultures with 95% ethanol (5 ml/plate) (Fukuzawa et al., 2008). The ethanolic solution was incubated under gentle shaking on a TE143® shaker table for 24 h, after which it was placed in SPEEDVARS AG 22331 for drying (Hamburg Concentrator 5301®). The final crude ethanolic extract (Ext-Ta) was solubilized in RPMI 1640 medium (Sigma).

The Ext-Ta was centrifuged at 4°C for 20 min, 2,370 × *g*, and the supernatant was centrifuged using a Centricon membrane (10-kDa pore) at 4°C for 15 min, 2,370 × *g* until reduction to 1 ml to obtain a high-molecular-weight fraction (HMWF). The eluate was separately collected to obtain a low-molecular-weight fraction below (LMWF) 10 kDa, and then the volume of the two fractions was adjusted to perform the bioassay.

### Parasites

*Leishmania amazonensis* (IFLA/BR/1967/PH8) were cultivated at 25°C and maintained in RPMI 1640 medium (Sigma) supplemented with 10% fetal bovine serum (FBS, Invitrogen-Gibco®) and 1 mg/ml of penicillin/streptomycin (Schering-Plow, Rio de Janeiro, Brazil). The promastigote forms of the protozoan were used in the stationary phase of growth.

### Macrophages

The macrophages J774 were cultured in RPMI 1640 medium (Invitrogen-Gibco®) supplemented with 10% of FBS and 1 mg/ml penicillin/streptomycin (Schering-Plow, Rio de Janeiro, Brazil), at 37°C with 5% of CO_2_ saturation. After cell confluence and monolayer formation, the cells were removed from the bottles using trypsin (Gibco®) and counted on a Neubauer hemocytometer chamber for the biological assays.

### Treatment With Ext-Ta, HMWF, and LMWF in *L. amazonensis* Promastigotes and Macrophages

The MTT cell viability assay was performed to investigate the cytotoxic effects of Ext-Ta, HMWF, LMWF, and pentamidine on *L. amazonensis* promastigotes and HMWF and LMWF on J774 macrophages. Parasites of 5 × 10^5^ per well in 96-well tissue plates were treated with different concentrations (0.31–4.95 ng/μl) of Ext-Ta and pentamidine (positive control) or HMWF and LMWF fractions (2.5–40 ng/μl) for 24 and 48 h. The non-infected J774 macrophages (1 × 10^5^) were seeded in 96-well tissue plates and treated with different fraction concentrations ranging from 2.5 to 40 ng/μl for 24 h. After that time, the samples were incubated with 5 mg/ml MTT for 4 h under the same culture conditions as described (Mosmann, 1983). After the reaction, DMSO (Sigma-Aldrich) was added to dissolve the salt crystals formed by mitochondrial metabolism, and the absorbance was determined at 570 nm in an ELISA reader (Molecular Devices, Sunnyvale, California). The obtained values were used to determine IC_50_.

### Phagocytosis Assays

The J774 macrophages (5 × 10^5^ cells per well) were cultivated in 24-well plates containing 13-mm round coverslips. The cells were incubated with 200 μl of RPMI 1640 medium supplemented with 10% FBS for 2 h, for adherence. The macrophage monolayer was washed with sterile phosphate-buffered saline (PBS) to remove non-adherent cells and was infected with promastigote forms of *L. amazonensis* (10:1) for 4 h at 37°C and 5% CO_2_. After the interaction, the plate was washed using RPMI medium to remove free parasites. The infected macrophages were treated with 2.5 ng/μl of the LMWF and incubated for 24 h. The cells were stained with hematoxylin–eosin in order to establish the percentage of infected macrophages by direct counting of 100 cells per slide under a light microscope, according to the following formula:

$$\begin{array}{l} \text{Phagocytic index}=\frac{\text{No. of amastigotes in }100\text{ macrophages}}{100\text{ macrophages}} \end{array}$$

### Transmission Electron Microscopy (TEM)

Promastigotes of *L. amazonensis* (5 × 10^5^ parasites per well) were treated for 24 h with different concentrations of LMWF ranging from 2.5 to 20 ng/μl. Macrophages (1 × 10^6^ cells per well) were infected with *L. amazonensis* (1:10 ratio) and treated for 24 h with 2.5 ng/μl LMWF. After washing with sterile PBS, the wells were fixed with 2.5% glutaraldehyde (SIGMA) in 0.1 mol sodium cacodylate buffer, at pH 7.2 for 1 h and postfixed in 1% osmium tetroxide and 0.8% potassium ferrocyanide (SIGMA). The samples were washed and dehydrated in an acetone series and embedded in a Polybed resin, which was polymerized at 60°C for 72 h (Vannier-Santos and Lins, 2001). Ultrathin sections were contrasted with 7% uranyl acetate and 4% lead nitrate aqueous solutions and observed on an FEI Morgagni 268 TEM.

### Statistical Analyses

The obtained data were expressed as mean ± standard deviation and analyzed in GraphPad Prism software version 5.0. The tests used included one-way ANOVA followed by Tukey posttest and the Student *t* test. IC_50_ was assayed by non-linear regression analysis. Values of *p* < 0.05 were considered significant. All experiments were performed in at least three independent assays in triplicate.

## Results

### *Trichoderma asperelloides* Ethanolic Extract and Its Fractions Inhibit the Proliferation of *L. amazonensis* Promastigotes

**Figure 1:**
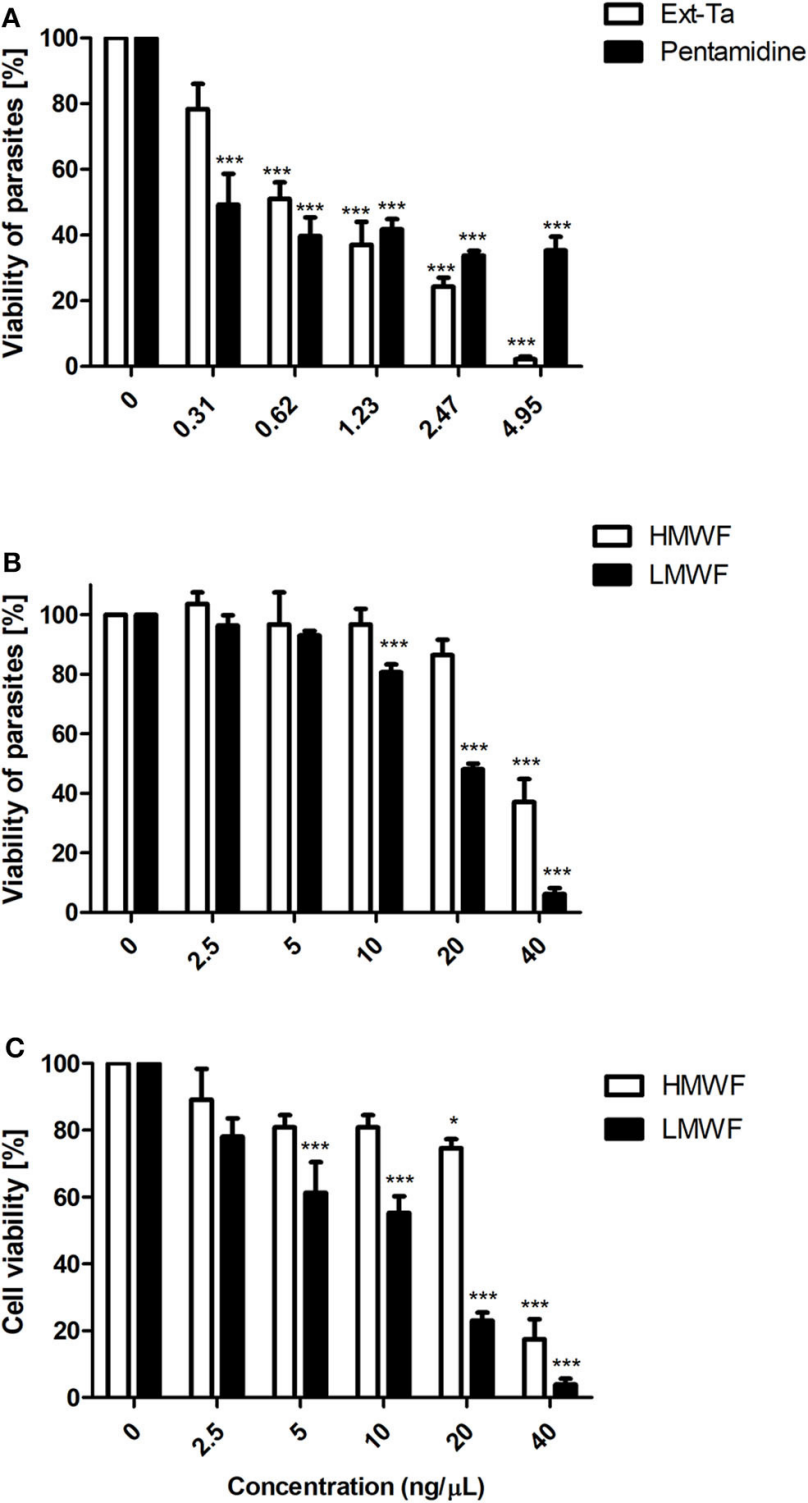
Ext-Ta treatment decreased *L. amazonensis* promastigote viability. **(A)** Promastigotes of *L. amazonensis* were treated with crescent concentrations of Ext-Ta and pentamidine and **(B)** different concentrations of HMWF and LMWF for 24 h. **(C)** J774 macrophages were treated with crescent concentrations of HMWF and LMWF for 24 h. The cell viability was performed by MTT assay. A value of *p* < 0.05 was considered for statistical significance. One-way ANOVA followed by Tukey post-test was performed to establish the statistical significance between the treatments in relation to the control. Ext-Ta: ethanolic extract of *T. asperelloides*; HMWF: high-molecular-weight fraction; LMWF: low-molecular-weight fraction; 0: control. *p < 0.05; ***p < 0.001.

The antileishmanial action of Ext-Ta and its HMWF and LMWF fractions in *L. amazonensis* promastigotes was dose dependent (*p* < 0.05). In the treatment with Ext-Ta, for 24 h, the parasite viability was reduced by 30–90% at concentrations ranging 0.31–4.95 ng/μl (Figure 1A), similarly to that in treatment for 48 h (Supplementary Figure 1A). The IC_50_ values of Ext-Ta were 1.09 and 1.53 ng/μl for 24 and 48 h, respectively. The viability of pentamidine-treated promastigotes was reduced by about 50%, in all concentrations tested, presenting the calculated IC_50_ value of 0.77 ng/μl (Figure 1A). The HMWF showed a significant reduction (*p* < 0.05) in the viability of the parasites only at the 40 ng/μl concentration as compared to the control (Figure 1B). LMWF produced a reduction of the parasite viability at the concentration of 10 ng/μl, which was significant (*p* < 0.05) in comparison to the control samples (Figure 1B), similarly to that of treatment for 48 h (Supplementary Figure 1B). The IC_50_ values observed for HMWF were 22.89 and 20.76 ng/μl, for 24 and 48 h, respectively, and those for LMWF were 18.16 and 12.85 ng/μl for 24 and 48 h, respectively.

The higher concentrations of HMWF and LMWF provoked around 80% and 90% of inviability in macrophages, respectively. LMWF showed a significant dose-dependent effect from 5 ng/μl in macrophages. The apparent IC_50_ value of HMWF was 23.28 ng/μl, and that of LMWF was 12.57 ng/μl (Figure 1C). LMWF was not toxic to macrophages or to *L. amazonensis* promastigotes at the concentration of 2.5 ng/μl. However, in the light microscopy analysis, it was observed that this concentration decreased promastigote motility (data not shown). This observation encouraged us to investigate whether LMWF interferes in the ultrastructure and proliferation of both evolutive forms of *L. amazonensis*.

### LMWF Alters the Ultrastructure of *L. amazonensis* Promastigotes and Intracellular Amastigotes

**Figure 2:**
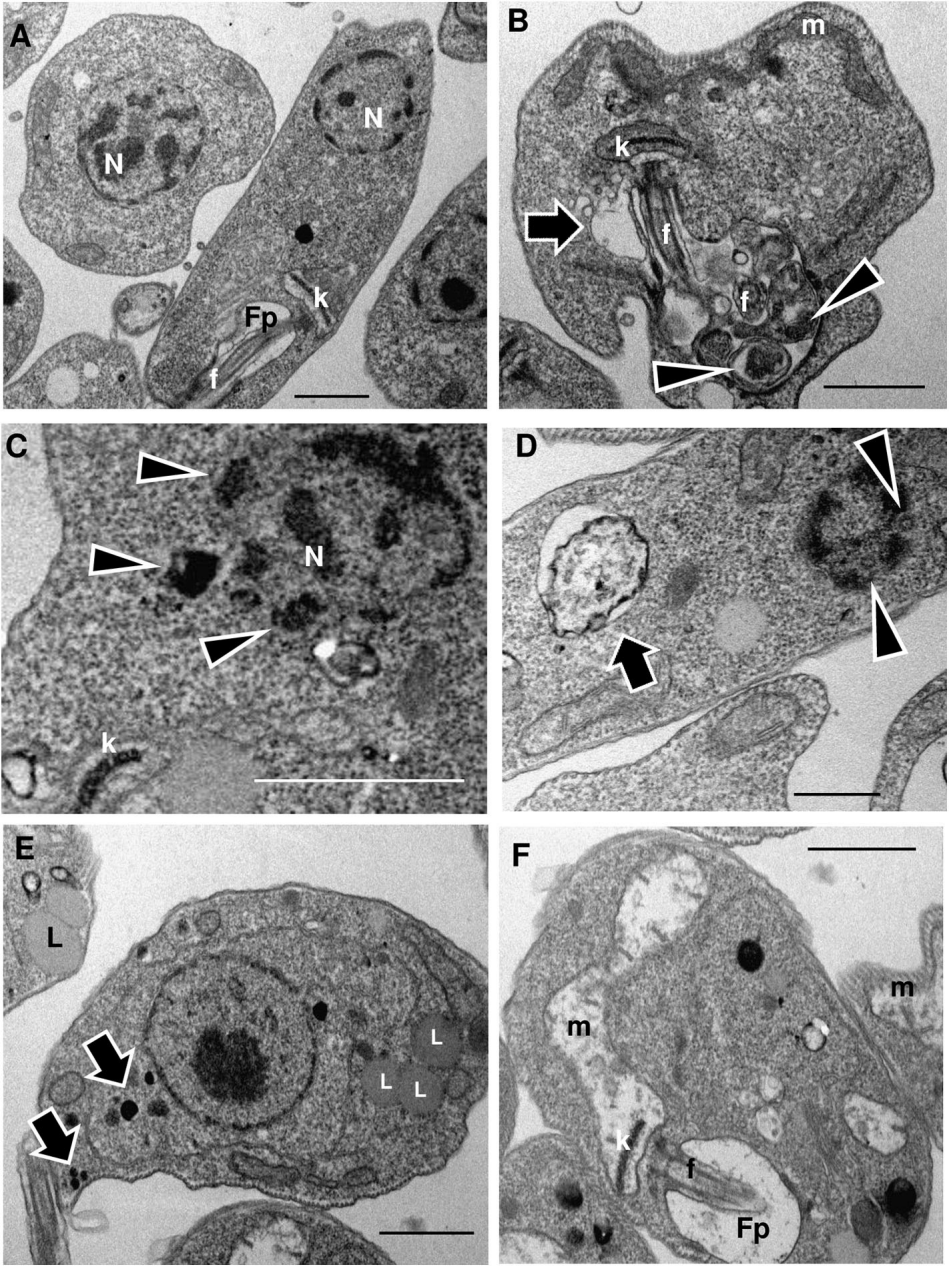
LMWF of Ext-Ta induces ultrastructural changes on *L. amazonensis* promastigotes. Promastigotes were treated with different concentrations of LMWF of Ext-Ta for 24 h, and the ultrastructure was analyzed by transmission electron microscopy. **(A)** Untreated control protozoan displaying the normal organization of the parasite cells; **(B)** promastigotes treated with 2.5 ng/μl, **(C)** 5 ng/μl, **(D)** 10 ng/μl, and **(E,F)** 20 ng/μl of LMWF. **(B)** Flagellar pocket presenting exosomes containing cytoplasmic material (arrowheads) enlarged contractile vacuole (arrow); **(C)** karyorrhexis (nuclear disruptor) with dense peripheral chromatin in direct contact with the cytoplasm (arrowheads); **(D)** parasite showing large autophagosome (arrow) and karyorrhectic nucleus displaying nuclear pores in direct contact with the cytoplasm (arrowheads); **(E)** Ext-Ta-treated parasites usually displayed numerous lipid bodies (L) and acidocalcisomes (arrows); **(F)** parasites presenting destroyed mitochondria (m). Fp, flagellar pocket; k, kinetoplast; L, lipid bodies; m, mitochondria; n, nucleus.

We observed that promastigotes treated with increasing LMWF concentrations exhibited significant ultrastructural changes as compared to the control (Figure 2A). An increase in contractile vacuole was observed, as well as a dilatation of the flagellar pocket presenting luminal exosomes displaying cytoplasmic contents (Figure 2B). Some LMWF-treated parasites presented nuclear disruption or karyorrhexis (Figures 2C,D), with dense peripheral chromatin (**C**) and nuclear pores (**D**) in direct contact with the cytoplasm. Large autophagosome (Figure 2D) and enhanced formation of acidocalcisomes and lipid bodies were also observed (Figure 2E) along with mitochondrial destruction (Figure 2F).

**Figure 3:**
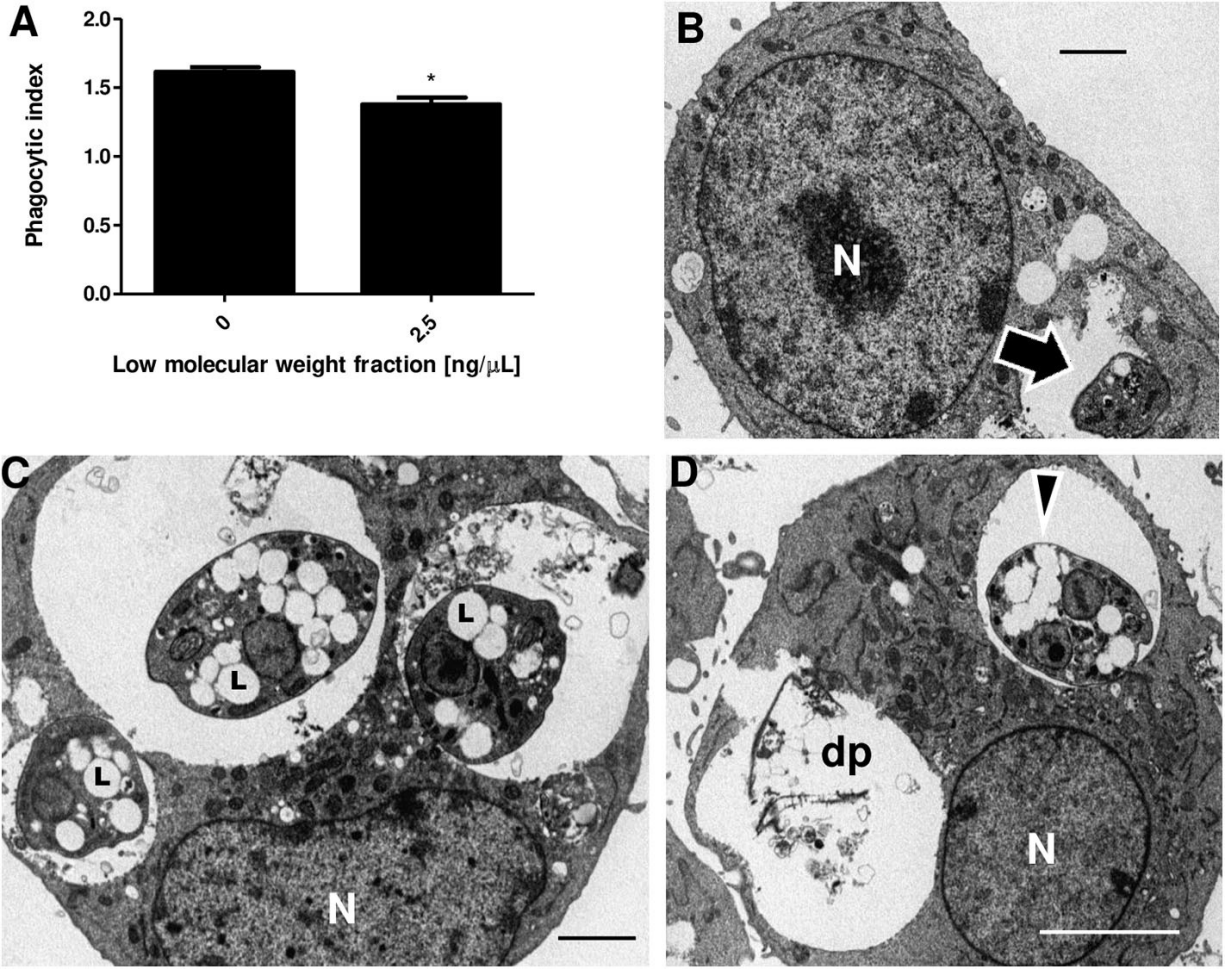
LMWF of Ext-Ta reduces parasite survival and causes ultrastructural changes on *L. amazonensis* amastigotes. **(A)** Phagocytic index of macrophages infected with *L. amazonensis* promastigotes and treated or not with 2.5 ng/μl of LMWF for 24 h. 0: control (untreated parasites). A value of *p* < 0.05 was considered for statistical significance; *p < 0.05 by comparison with Student *t* test. **(B)** Untreated culture of macrophages infected with *L. amazonensis* promastigotes (10:1 ratio) presenting intact amastigote inside a parasitophorous vacuole (arrow). **(C,D)** Macrophages infected and treated with 2.5 ng/μl of LMWF for 24 h showed parasites with numerous lipid bodies (**C**, L) which eventually coalesced, reaching the parasite cell surface (**D**, arrowhead). **(D)** The necrosis of the parasites culminated in the complete destruction of parasites (dp). N, macrophage nucleus.

The phagocytic index obtained by counting infected cells and the number of parasites inside the macrophages was significantly different between untreated J774 cells (1.61) and cells treated with 2.5 ng/μl of LMWF (1.38) (Figure 3A). To investigate if LMWF also affects the ultrastructure of *L. amazonensis* amastigotes, infected macrophages were treated with 2.5 ng/μl of LMWF and compared to amastigotes grown on untreated macrophages (Figure 3B). LMWF caused the biogenesis of numerous lipid bodies (Figure 3C), which coalesced in a cumulative manner, eventually reaching the parasite cell surface (Figure 3D) and dead intracellular parasites apparently by necrosis (Figure 3D).

## Discussion

The bioactive compounds of endophytic fungi such as *Trichoderma* sp. have a potential industrial application with antimicrobial, antiviral, antioxidant, and immunomodulatory activities. These compounds include alkaloids, glycosides, flavonoids, phenolic acid, quinones, xanthones, terpenoids, and steroids, among others (Shukla et al., 2014). The discovery of new compounds and strategies against pathogenic microorganisms can be a hope to treat a range of diseases, especially neglected diseases in undeveloped countries.

We have previously shown that an *in vivo* treatment of cerebral malaria with the ethanolic extract of *Trichoderma stromaticum* has promising effects, including reduction of parasitemia and inflammation, increased survival, and prevention of neurological signs of cerebral malaria in C57BL/6 mice (Cariaco et al., 2018). In a similar way, the use of crude ethanolic extracts of *T. asperelloides* and its fraction can efficiently inhibit *Staphylococcus* growth and biofilm formation, representing a candidate for the development of new antibiotics against gram-positive bacteria (Santos et al., 2018).

The data obtained in this study have shown for the first time that the *T. asperelloides* fungus has a leishmanicidal action against *L. amazonensis*. We observed that the ultrastructure of the promastigotes and amastigotes was affected by the treatment with LMWF and that both the Ext-Ta and LMWF obtained from the extract have potent toxic activity against promastigote forms of *L amazonensis*.

The treatment for leishmaniasis is currently toxic, limited, and long. New perspectives for treatment need to be made. The IC_50_ of the ethanolic extract of *T. asperelloides* for promastigotes was shown to be 6-fold lower than that observed in studies using the endophytic fungus *Cochliobolus* sp., the plant *Vernonia polyanthes*, and secondary metabolites of the fungus *Eurotium repens* (Campos et al., 2008; Gao et al., 2012; do Nascimento et al., 2015), demonstrating that products of this fungus have technological potential for developing novel leishmanicidal agents.

TEM has been frequently used to elucidate ultrastructural changes in protozoa submitted to compounds with leishmanicidal action (Rodrigues and de Souza, 2008; Vannier-Santos and De Castro, 2009). Here, we observed important changes in the parasite organization which may shed light on the mechanisms of action of the Ext-Ta fraction.

The triggering of karyorrhexis or nuclear disruption is associated with both apoptosis and necrosis cell death pathways (Cheville, 2009). Karyorrhexis was reported in late apoptosis of human carcinoma cells treated with nanoparticles presenting fungal filtrates of *Pestalotiopsis microspora* (Netala et al., 2016). Karyorrhexis was also associated with necrosis induced by acetaminophen (Jacob et al., 2007).

Exosomes with cytoplasmic core within the flagellar pocket and autophagosome formation were previously reported in *L. amazonensis* promastigotes treated with 22,26-azasterol (Rodrigues et al., 2002), and multivesicular bodies were also detected in this site following starvation-induced autophagy in *Leishmania major* (Basmaciyan et al., 2018).

Interestingly, the formation of acidocalcisomes and lipid bodies was also associated with ergosterol biosynthesis inhibitor-induced autophagy (Vannier-Santos et al., 1995, 1999; Rodrigues et al., 2002). Lipid body accumulation is also related to autophagy (Singh et al., 2009). Furthermore, lipid accumulation plays different roles in the non-apoptotic regulated cell death pathways of necroptosis and ferroptosis (Magtanong et al., 2016).

Nowadays, acidocalcisomes are seen as lysosome-related organelles, required for autophagy in *T. brucei* (Li and He, 2014) and proposed to act cooperatively with contractile vacuoles in protozoan parasite osmoregulation (Docampo et al., 2010). In this regard, trypanocidal compounds such as squalene synthase inhibitors may also cause contractile vacuole enlargement (Braga et al., 2004). It remains to be determined whether the osmoregulatory mechanism could be involved in cell death mechanism triggered by fungal derivatives, but calcium homeostasis is involved in the mode of action of antiparasitic agents on *Leishmania donovani* (Pinto-Martinez et al., 2018) and *Trypanosoma cruzi* (Benaim et al., 2020). Lipid body formation may comprise an escape mechanism as this compartment is an intracellular site of synthesis of prostaglandins, such as PGF_2a_, inhibiting different facets of the immune response (Vallochi et al., 2018).

The mitochondrial swelling and total extraction of the matrix content were observed in the *L. amazonensis* promastigotes treated with the LMWF. Based on ultrastructural observations, we found that a higher concentration of the compound affected the mitochondrial ultrastructure through extensive swelling and severe damage to internal membranes (Fonseca-Silva et al., 2015), similar to what has been observed for *L. amazonensis* promastigotes treated with different concentrations of LMWF.

The most frequent effect of chemotherapeutic agents on trypanosomatid mitochondria is mitochondrial dilation, indicative of a dysfunction in membrane potential. Studies indicate that this organelle undergoes a change in its lipid organization and ultrastructure, in trypanosomatids like *T. cruzi* and *Leishmania* spp., when the parasites are incubated with sterol biosynthesis inhibitors, considering that ergosterol is an important structural element present in mitochondrial membranes (Adade and Souto-Padrón, 2010; Medina et al., 2012), possibly modulating the organelle membrane stability and fusogenicity (Vannier-Santos et al., 1995). It was demonstrated that alterations or damage to the mitochondria of *L. amazonensis* may be involved in the death of the parasite by apoptosis due to changes in the mitochondrial membrane potential, associated with an increase in mitochondrial reactive oxygen species (ROS) production and decreased detoxification by the parasite (Garcia et al., 2013).

The crude extract of *T. asperelloides* was shown to be toxic to *L. amazonensis*. The impairment in parasite survival appears to be associated with the ultrastructural changes caused by the LMWF in both developmental forms. The amastigote forms of *L. amazonensis* appear to be more sensitive than promastigote ones due to the lower concentration of the LMWF and significant ultrastructural changes including nuclear disruption, leading to parasite cell death.

Due to the toxic and often inefficient therapy of leishmaniasis, the discovery of new effective, safe, and low-cost bioactive compounds is urgently required. Thus, *Trichoderma* spp. may comprise a promising fungus with the potential to develop novel chemotherapy tools for this disease. Preliminary data from our group demonstrated the presence of alkaloids as one of the major components of the LMWF of *T. asperelloides* (Supplementary Figure 2).

According to Scotti et al. (2016), alkaloids are secondary metabolites with several useful pharmacological properties for the treatment of malaria and bacterial infections, in addition to having an excellent antileishmanial activity, with effects on the biology of the parasite (Mishra et al., 2009). Previous studies report that natural products such as alkaloids have an inhibitory activity against the enzyme arginase of *Leishmania* sp. (Manjolin et al., 2013; Lacerda et al., 2018). This enzyme has its importance both in the polyamine biosynthesis, essential for the biology of the parasite (Vannier-Santos and Suarez-Fontes, 2017), and in the evasion of the host's immune response against the parasite (D'Antonio et al., 2013). In this regard, inhibition of polyamine metabolism can cause mitochondrial destruction and formation of flagellar pocket exosomes in *L. amazonensis* (Vannier-Santos et al., 2008). Thus, this polycation metabolism may comprise a promising drug target for the development of leishmanicidal compounds (Cruz et al., 2013; Maquiaveli et al., 2016; Roberts and Ullman, 2017).

Regardless of the cell toxicity observed at certain concentrations, the leishmanicidal activity found in this fungus could be used for the development of new drugs for local treatment of lesions, thus avoiding systemic side effects. The development of an affordable simple drug with easy administration would be an asset for the local treatment of cutaneous leishmaniasis. Future plans involve a further study of the active components of this fungus, with improved identification techniques, in addition to understanding the role of these compounds, especially alkaloids, in the biology of the parasite.

## Data Availability Statement

All datasets generated for this study are included in the article/Supplementary Material.

## Author Contributions

DL, JL-S, and CP conceived and designed the experiments. DL, DA, LS, VP, TC-G, JL-S, and CP generated and analyzed the data. IS-J and CP contributed reagents. MV-S analyzed the TEM. DL, US, DA, IS-J, TC-G, and MV-S drafted the manuscript. All authors contributed to manuscript revision, read, and approved the submitted version.

## Conflict of Interest

The authors declare that the research was conducted in the absence of any commercial or financial relationships that could be construed as a potential conflict of interest.
