# Supplementary material for: Ethanolic Extract of the Fungus Trichoderma asperelloides Induces Ultrastructural Effects and Death on Leishmania amazonensis
Source: Front Cell Infect Microbiol. 2020 Jul 15;10:306. doi: 10.3389/fcimb.2020.00306 (PMC7373754; doi:10.3389/fcimb.2020.00306)
Supplement: Supplementary file 1 [file Image_1.pdf]

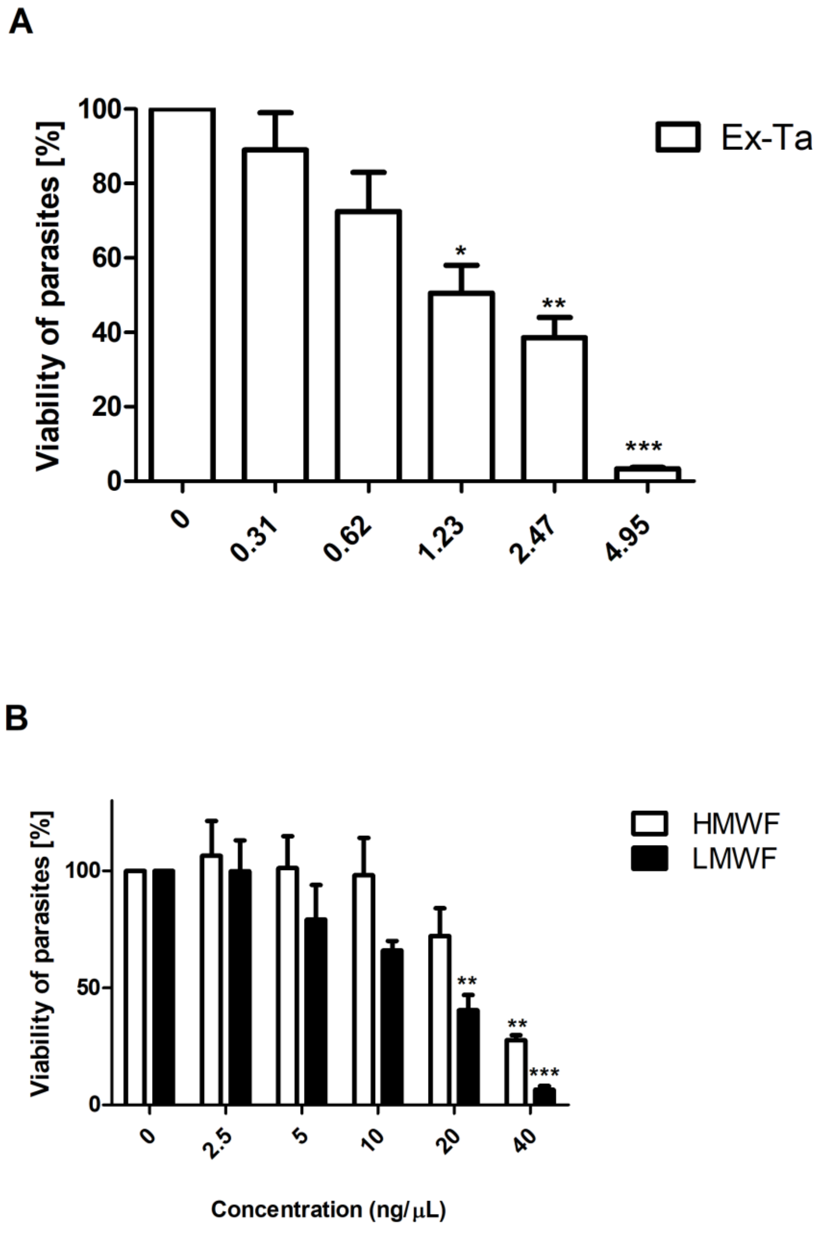

Supplementary Figure 1. Ext-Ta treatment decreased *L. amazonensis* promastigotes viability. (**A**) Promastigotes of *L. amazonensis* were treated with crescent concentrations of Ext-Ta and (**B**) different concentrations of HMWF and LMWF for 48h and the viability was performed by MTT assay. Value of $p < 0.05$ was considered for statistical significance. One-way ANOVA followed by Tukey post-test were performed to establish the statistical significance between the treatments in relation to the control. **Ext-Ta:** ethanolic extract of *T. asperelloides*; **HMWF:** high molecular weight fraction; **LMWF:** low molecular weight fraction; **0** - Control. $^{*}p < 0.05$; $^{**}p < 0.01$; $^{***}p < 0.001$.
