# Supplementary material for: Ethanolic Extract of the Fungus Trichoderma asperelloides Induces Ultrastructural Effects and Death on Leishmania amazonensis
Source: Front Cell Infect Microbiol. 2020 Jul 15;10:306. doi: 10.3389/fcimb.2020.00306 (PMC7373754; doi:10.3389/fcimb.2020.00306)
Supplement: Supplementary file 3 [file Table_1.DOCX]

Supplementary Material

# Supplementary Materials and Methods

**1.1 Identification of components of the low molecular weight fraction of *Trichoderma. aperelloides***

The low molecular weight fraction was washed with methanol in order to extract the constituents soluble in organic solvent, and thus be able to analyze them by thin layer chromatography (CCD) on silica gel.

The methanolic solution obtained from this extraction was analyzed to qualitatively determine the presence of alkaloids using Dragendorff's reagent (solution of bismuth subnitrate with potassium iodide in acetic acid (ALICE et al., 1995)). In this analysis, the sample, soluble in methanol, was applied on a CCD plate, and eluted with methanol. After elution, the plate was sprayed with Dragendorff's reagent.

Qualitative analysis was also performed to determine the presence of flavonoids in the extracted fraction, using the NP (diphenylboryloxyethylamine in methanol) reagent (WAGNER and BLADT, 2001). In this analysis, the sample was applied in CCD and eluted with methanol. After drying, the plate was sprayed with the reagent and analyzed by irradiating ultraviolet light (365 nm).

# Supplementary Results

In thin layer chromatography on silica gel, it was possible to observe the presence of alkaloids, due to the appearance of orange spots on the surface of the plate. However, was not verified the appearance of fluorescent spots that would indicate a positive result for flavanoids (**Supplementary Figure 2**).

# Supplementary Figures Legends

**Supplementary Figure 1.** **Ext-Ta treatment decreased *L. amazonensis* promastigotes viability.** (**A**) Promastigotes of *L. amazonensis* were treated with crescent concentrations of Ext-Ta and (**B**) different concentrations of HMWF and LMWF for 48h and the viability was performed by MTT assay. Value of *p* < 0.05 was considered for statistical significance. One-way ANOVA followed by Tukey post-test were performed to establish the statistical significance between the treatments in relation to the control. **Ext-Ta:** ethanolic extract of *T. asperelloides*; **HMWF:** high molecular weight fraction; **LMWF:** low molecular weight fraction; **0** - Control. **p* < 0.05; ^**^*p* < 0.01; ^***^*p*< 0.001.

**Supplementary Figure 2.** **LMWF contains alkaloids.** Preliminary identification of alkaloids (black circle) was through thin layer chromatography on silica gel.

# Supplementary References

Alice, B.C.; Siqueira, N.C.S.; Mentz, L.A.; Silva, G.A.A.B.; José, K.F.D. (1995). Metodologia de análise de Plantas. *Plantas medicinais de uso popular: Atlas farmacognóstico.* Canoas: Editora ULBRA, 11-19.

Wagner, H.; Blad, S. (2009). Plant Drug Analysis: a thin layer chromatography atlas. *Springer*. 2^nd^ ed. (reprint).
